# Association between smartphone overdependency and mental health in Korean adolescents during the COVID pandemic; Age-and gender-matched study

**DOI:** 10.3389/fpubh.2022.1056693

**Published:** 2022-12-21

**Authors:** Na-Hye Kim, Jae-Moo Lee, Seo-Hyung Yang, Jung-Min Lee

**Affiliations:** ^1^Department of Taekwondo, Kyung Hee University, Yongin-si, South Korea; ^2^College of Sport Science, Sungkyunkwan University, Suwon-si, South Korea; ^3^School of Global Sport Studies, Korea University, Sejong-si, South Korea; ^4^Sports Science Research Center, Kyung Hee University, Yongin-si, South Korea; ^5^Department of Physical Education, Kyung Hee University, Yongin-si, South Korea

**Keywords:** adolescent, smartphone dependency, subjective health status, stress, happiness

## Abstract

**Purpose:**

This study aimed to examine the relationship between smartphone dependency (SD) and mental health (MH) in adolescents in order to develop and implement plans pertaining to SD control.

**Methods:**

Raw data from the 16th Online Adolescent Health Behavior Survey in 2020 were analyzed. A total of 482 respondents were selected as study subjects based on their experience of smartphone overdependence (SO), specifically, 241 participants whose score for SO was 37 or higher (Group 2) and age- and gender-matched 241 participants whose score was lower than 10 (Group 1).

**Results:**

Frequency analysis, cross-tab analysis (χ^2^ test), and multinomial logistic regression were performed Analysis shows that the MH affecting the increase in SO is the subjective perception of happiness, subjective perception of stress, sadness and despair, and experience of Loneliness. But, the variable affecting the reduction is the subjective evaluation of sleep quality. The likelihood of SO increased as adolescents felt unhappier [Exp (*β*) = 2.408] and more stressed [Exp (*β*) = 4.453] and more often felt lonely [Exp (*β*) = 8.149], but the likelihood decreased as they had neither sufficient nor insufficient sleep duration [Exp (*β*) = 0.344]. The findings suggest that it is necessary to develop aggressive measures for the prevention and management of MH in adolescents showing SO because mental health is closely linked to SD. In developing the measures, realistic approaches to widely pervasive SO among adolescents should be explored by taking into account MH factors, that is, predictors of SO, and the characteristics of youths, such that they can self-control smartphone use and form desirable life habits.

## Introduction

Smartphones are used in daily life, from the public domain (e.g., work) to the private one (e.g., shopping and conversations with friends). Smartphone use is steadily rising as it is an essential tool in modern society have practical and convenient benefits by connecting to the internet and downloading diverse apps and is becoming more socially acceptable. More than half of the global population use smartphones (1, 2), and particularly among children and adolescents, the rate of smartphone adoption is rapidly increasing (3, 4).

In today's world, it is difficult to imagine life without smartphones. Smartphone overdependence refers to a state in which individuals experience interpersonal conflict, physical discomfort, and difficulties in the family, school, and workplace, because they cannot self-control smartphone use (5). Smartphone overdependence can lead to cognitive problems (6) and pathological conditions, such as low sleep quality (7). Accordingly, many individuals want to reduce their use of smartphones (8) but only about half of them are successful (9), showing that smartphone addiction is so strong (7, 10). It has even become one of the public health issues in many countries worldwide (11).

Unlike previous generations, adolescents have less experience in analog media. They are defined as the young generation who have grown up with various IT systems from birth. They have used smartphones since childhood and are used to obtaining information, making new friends, and expressing themselves through smartphones. Adolescents take smartphones for granted and see the smartphone as the main communication tool at the center of their lives (12). Thus, they are highly attached to smartphones, tend to consider smartphones as a part of themselves, and be dependent on them. Of course, there are benefits of using smartphones, such as convenience in daily life, educational use, and stress reduction. However, smartphone overdependence during adolescence, which is a critical phase in the lifespan, can result in psycho-emotional pathology that cannot be ignored (13). According to previous studies, adolescents showing smartphone overdependence may experience various negative health outcomes, such as depression, sleep disturbances, low academic achievement, physical problems (i.e., text neck), and social withdrawal (14–16). In particular, social distancing and an increase in remote learning due to the prolonged COVID-19 pandemic have made it much easier for adolescents to have access to smart media and smartphones. Hence, adolescent smartphone overdependence may expand to an alarming degree. The problem of adolescent smartphone overdependence can manifest itself as an issue in society at large, as the problem may continue into adulthood. Therefore, society should take intervention measures to prevent smartphone overdependence so that adolescents in the phase in which self-awareness and the attitude toward life are formed can grow physically and mentally while maintaining a healthy lifestyle.

Various measures to treat smartphone addiction are being introduced, but have failed to reduce the dependency rate effectively because they are vulnerable to this information and difficult to access (17). It is possible to intervene to decrease smartphone dependency at school or home superficially, but it is not easy for adolescents by themselves to perceive overdependence as a problem. Even if they do, it is difficult to change their behavior unless they motivate themselves to address the problem. In other words, an effective preventive or treatment approach does not yet exist (18). The reason is that an environment has already been created in which it is difficult to carry out daily living tasks without an internet-accessible smartphone, which is essentially addictive (7, 10). Therefore, it is critical to control the level of smartphone dependency such that adolescents can use them correctly and appropriately. Accordingly, it is important not only to identify the causes of smartphone overdependence but also to investigate a mechanism for its prevention.

This study was conducted to examine the pattern of association between smartphone dependency and mental health in adolescents to develop plans for smartphone dependency control and health promotion. Therefore, this study aimed to systemically investigate factors associated with smartphone overdependence, to explore the ways to ameliorate the problems early, to help develop a correct understanding of adolescent smartphone dependency, and to develop effective preventive measures for future effective interventions.

## Materials and methods

### Participants

This study analyzed raw data from the 16th Online Korean Youth Risk Behavior Web-based Survey, a national statistical survey (approval number, 117058) performed by the Korea Disease Control and Prevention Agency, Ministry of Health and Welfare of South Korea, and Ministry of Education, Science, and Technology based on the National Health Promotion Act (Article 19) to monitor primary health behavior among Korean adolescents. Participants conducted the anonymous web-based survey voluntarily and completed the self-administered during a regular class period. The survey was distributed to a total of 59,925 Korean adolescents between August and November 2020 and of those, 54,948 from 793 schools responded (response rate: 94.9%).

In 2020 survey consisted of 120 questions assessing the demographic information and 15 areas of health-related behaviors and the smartphone dependency screening question was newly included in the 2020 survey.

As a result of analyzing the smartphone dependence of a total of 45,291 subjects excluding 9,657 who answered errors to the questions related to this study, there were 34,411 (75.98%) in the general user group (a score of 22 or lower), 9,740 (21.51%) in the potential risk group (a score between 23 and 30), and 1,140 (2.52%) in the high-risk group (a score of 31 or higher). However, considering that it became permissible for adolescents to use smartphones in their studies and daily life due to COVID-19, it was necessary to strictly review the dependence on smartphones of the subject to achieve the purpose of the research. Therefore, in this study, 241 adolescents (Group 1) in the top 0.53% of high-risk groups (more than 37 points) were selected, and out of 7,925 teenagers (10 points, 17.50%) who said they had little dependence on smartphones among general users, 241 adolescents were randomly selected by matching gender and age with adolescents in Group 1, and a total of 482 people were used for analysis. Approval from Kyung Hee University IRB was not sought because the study used publicly available raw data.

### Study variables

The following variables were analyzed to examine mental health in adolescents spending too much time on smartphones: the experience of smartphone overdependence; subjective health status; subjective perception of happiness; subjective perception of stress, sadness, and despair; subjective evaluation of sleep quality; and experience of loneliness. The details are as follows.

### Smartphone dependency

To estimate the rate of smartphone overdependence, the Smartphone Overdependence Scale was used. This scale was developed by the Korea National Information Society Agency in 2016. It consists of 10 items in the domains of salience, self-control failure, and serious consequences. The highest total score is 40 points. Smartphone dependency is classified based on total scores. A score of 22 or lower is classified as general use, a score between 23 and 30 as a potential risk, and a score of 31 or higher as a high-risk (19). In this study, however, considering the changes in society due to the COVID-19 pandemic and increased digitization, the smartphone non-dependent group has a score lower than 10 (general user group), and the smartphone overdependent group has a score of 37 or higher (high-risk group).

### Mental health

To examine mental health, variables regarding subjective health status; subjective perceptions of happiness and stress, sadness, and despair (depression); subjective evaluation of sleep quality; and the experience of loneliness were used. First, subjective health status was based on the response to “in general, how would you describe the condition of your health?” In the raw data, the answer choices were “excellent,” “good,” “fair,” “poor,” and “very poor.” In this study, the raw data were recoded into three categories “good,” “fair,” and “poor.” Second, the subjective perception of happiness was based on the response to “in general, how happy are you?” In the raw data, the answer choices were “very happy,” “somewhat happy,” “neutral,” “somewhat unhappy,” “and very unhappy.” In the study, the five answer categories were recoded into three categories “happy, “neutral,” and “unhappy.” Third, the subjective perception of stress was based on the response to “in general, how stressed are you?” In the raw data, there were five answer choices ranging from “I feel very stressed” to “I don't feel stressed at all.” In the study, the data were recoded into three categories of “very stressed,” “somewhat stressed,” and “not at all stressed.” Fourth, the subjective perception of sadness/despair (depression) was based on the response to “in the last 12 months, have you felt sad or despaired continuously for at least 2 weeks such that you could not carry out daily life activities?” Answer categories in the raw data, “yes” and “no,” were used in the study, as well. Fifth, the subjective evaluation of sleep quality was based on the response to “do you think the number of sleep hours in the last 7 days was sufficient for recovering from fatigue?” The raw data were coded with five answer categories ranging from “very sufficient” to “not sufficient at all,” and recoded data using three categories of “sufficient,” “neither sufficient nor insufficient,” and “insufficient” were used in the study. Sixth, the experience of loneliness was based on the response to “in the last 12 months, how frequently did you feel lonely?” The same answer categories in the raw data, that is, “never,” “rarely,” “sometimes,” “often,” and “always,” were used in the study.

### Data analysis

The participants' characteristics and anthropometric information were summarized by SPSS 25.0 version (SPSS Inc., Chicago, IL, USA). The participant's personal information (i.e., sex, age) was examined by descriptive statistics and anthropometrics information (i.e., height, weight, and BMI). Additional independent t-tests were also used to examine the difference in anthropometric information between the groups. The chi-square analysis (χ^2^ test) was utilized to investigate mental health factors' differences between smartphone-non-dependent and smartphone-overdependent groups, and a *p*-value was used to evaluate whether there are significant differences between the two groups. Additionally, multinomial logistic regression was used to demonstrate the association between smartphone dependency and other variables (i.e., Subjective health status, Subjective perception of happiness, Subjective perception of stress, Sadness, and despair, Subjective evaluation of sleep quality, Experience of loneliness) and results were presented as odds ratios (OR) with 95% confidence intervals (95% CI). Statistical significance was determined based on *p* < 0.05, in all analyses.

## Result

Participants' demographic characteristics (i.e., gender and age) and anthropometric measurements (i.e., height, weight, and BMI) are summarized as the frequency and proportions. The sample in the present study included 482 adolescents and the average age was 15.42 years; males were 48.3% and females were 51.7% in each group (i.e., smartphone non-dependent and smartphone overdependent group). The adolescents' characteristics (i.e., gender and age) indicated no significant differences among the two different groups because age and gender were matched for each group (*p* < 0.05). The findings discussed so far are presented in Table 1.

**Table 1:** Characteristics and anthropometrics for participants in two groups.

**Variables**		**Group 1 (*****n*** = **241)**		**Group 2 (*****n*** = **241)**	
		**No. (%)**	**Mean ±SD**	**No. (%)**	**Mean ±SD**
Gender	Male	117 (48.55)		116 (48.13)	
	Female	124 (51.45)		125 (51.87)	
Age (year)	12 yr	11 (4.56)		11 (4.56)	
	13 yr	26 (10.79)		26 (10.79)	
	14 yr	36 (14.94)		36 (14.94)	
	15 yr	52 (21.58)		52 (21.58)	
	16 yr	35 (14.52)		35 (14.52)	
	17 yr	56 (23.24)		56 (23.24)	
	18 yr	25 (10.37)		25 (10.37)	
Height (cm)	Male		171.86 ± 7.98		172.08 ± 8.64
	Female		161.33 ± 5.33		161.34 ± 5.11
Weight (kg)	Male		64.53 ± 14.23		65.87 ± 14.20
	Female		52.92 ± 8.56		53.90 ± 8.26
BMI (kg/m^2^)	Male		21.76 ± 4.16		22.17 ± 4.16
	Female		20.30 ± 2.88		20.68 ± 2.81

Group 1 = Smartphone non-dependent group (lower than 10 points); Group 2 = Smartphone overdependent group (37 points or higher); SD, standard deviation; BMI, body mass index. There are no significant differences in height, weight, and BMI between the early Group 1 and Group 2.

In the analysis of mental health variables according to the level of smartphone dependency, statistically significant between-group differences were found in all variables (*p* < 0.001). Regarding subjective health status, the proportion of “good” was the highest in both the smartphone overdependent and the non-dependent groups, 73.44 and 56.43%, respectively. The proportion of “poor” was low, 5.39 and 18.26% in the smartphone overdependent and non-dependent groups, respectively. Regarding the subjective perception of happiness, in the smartphone non-dependent group, the proportion of “happy” was high (70.12%) and that of “unhappy” was low (4.56%). On the other hand, in the smartphone-overdependent group, the proportions of “happy” and “neutral” were high, 36.93 and 35.68%, respectively. Regarding perceived stress, in the smartphone non-dependent group the proportion of “somewhat stressed” was high (39.00%), whereas in the smartphone overdependent group, the proportion of “very stressed” was high (67.22%) and that of “not stressed” low (6.64%). With regard to sadness/despair, the proportion of “no” answers was high (75.52%) in the smartphone non-dependent group, whereas the proportion of “yes” answers was high (56.85%) in the smartphone overdependent group. Regarding the subjective evaluation of sleep quality, in the smartphone non-dependent group, the proportions of “insufficient” (33.20%), “neither” (33.20%), and “sufficient” (33.61%) answers were evenly distributed. In the smartphone-overdependent group, the proportion of “insufficient” answers was the highest, 60.58%. Regarding the experience of loneliness, in the smartphone non-dependent group, the proportion of “never” answers was high (41.08%) and that of “always” answers was low (2.07%). By contrast, in the smartphone-overdependent group. The proportions of “sometimes” (25.83%), “always” (22.41%), and “often” (24.90%) answers were high at a similar level, and the proportion of “rarely” answers was low (11.62%). The findings discussed so far are presented in Table 2.

**Table 2:** Cross-tab analysis results on mental health variables by the group.

**Variables**		**Group 1 (*n* = 241)**	**Group 2 (*n* = 241)**	**Total (*n* = 482)**	***χ^2^***
Subjective health status	Poor	13 (5.39%)	44 (18.26%)	57 (11.83%)	23.123^***^
	Fair	51 (21.16%)	61 (25.31%)	112 (23.24%)	
	Good	177 (73.44%)	136 (56.43%)	313 (64.94%)	
Subjective perception of happiness	Unhappy	11 (4.56%)	66 (27.39%)	77 (15.98%)	68.344^***^
	Neutral	61 (25.31%)	86 (35.68%)	147 (30.50%)	
	Happy	169 (70.12%)	89 (36.93%)	258 (53.53%)	
Subjective perception of stress	Very	68 (28.22%)	162 (67.22%)	230 (47.72%)	86.317^***^
	Somewhat	94 (39.00%)	63 (26.14%)	157 (32.57%)	
	Not at all	79 (32.78%)	16 (6.64%)	95 (19.71%)	
Sadness and despair	Yes	59 (24.48%)	137 (56.85%)	196 (40.66%)	52.314^***^
	No	182 (75.52%)	104 (43.15%)	286 (59.34%)	
Subjective evaluation of sleep quality	Insufficient	80 (33.20%)	146 (60.58%)	226 (46.89%)	36.306^***^
	Neither sufficient nor insufficient	80 (33.20%)	48 (19.92%)	128 (26.56%)	
	Sufficient	81 (33.61%)	47 (19.50%)	128 (26.56%)	
Experience of loneliness	Always	5 (2.07%)	54 (22.41%)	59 (12.24%)	86.430^***^
	Often	25 (10.37%)	60 (24.90%)	85 (17.63%)	
	Sometime	71 (29.46%)	62 (25.73%)	133 (27.59%)	
	Rarely	41 (17.01%)	28 (11.62%)	69 (14.32%)	
	Never	99 (41.08%)	37 (15.35%)	136 (28.22%)	

Group 1; Smartphone nondependent group (lower than 10 points), Group 2; Smartphone overdependent group (37 points or higher);

*** *p* < 0.001.

**Figure 1:**
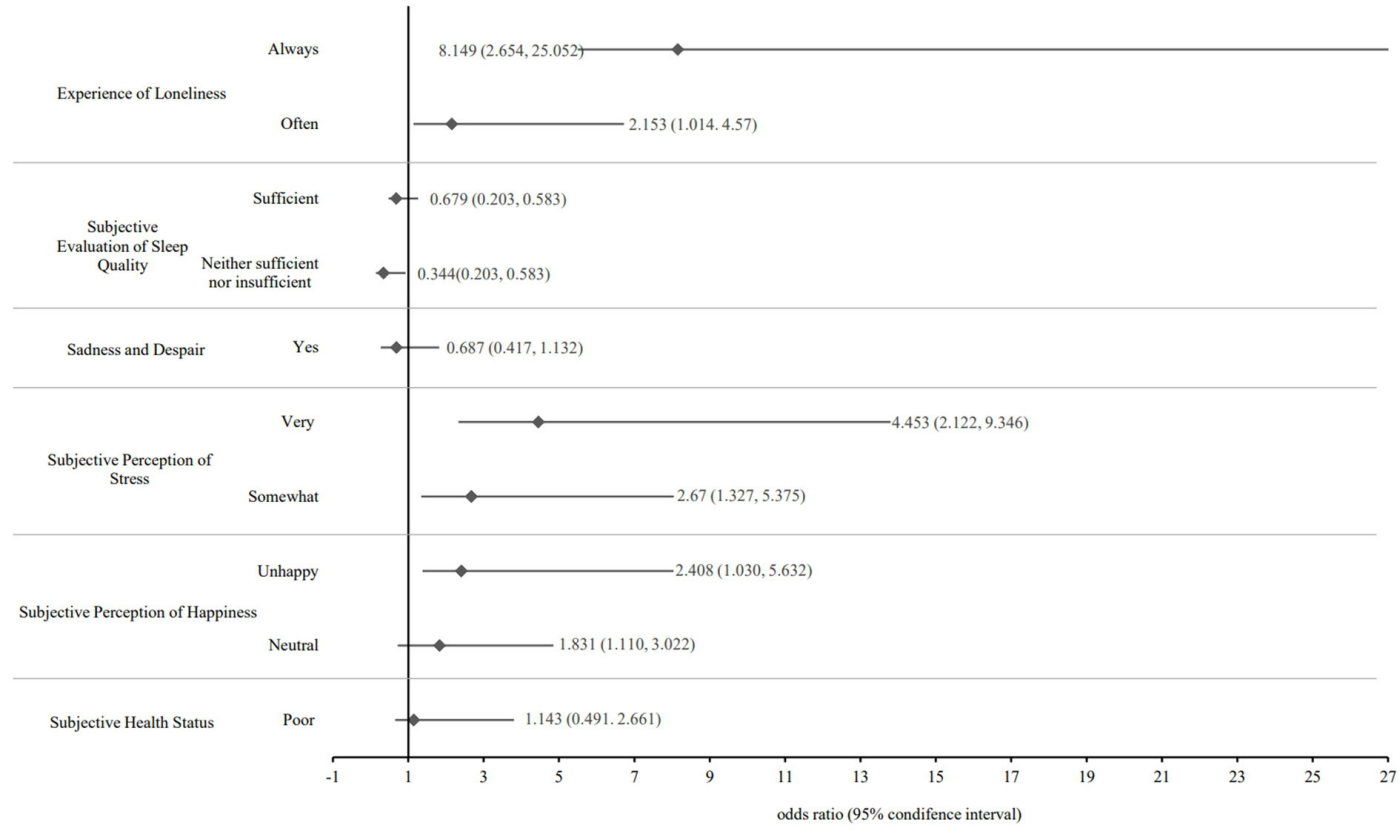
Forest plot of the odds ratios for mental health factors.

A multinomial logistic regression analysis was performed to identify factors influencing smartphone dependency in adolescents. The results are presented in Table 3. Smartphone overdependence was significantly associated with the subjective perception of happiness. Compared to being happy, being neutral was 1.831 times higher (OR = 1.831; 95% CI = 1.110, 3.022) and 2.408 times higher (OR = 2.408; 95% CI = 1.030, 5.632) for “unhappy.” Regarding the subjective perception of stress, the likelihood of smartphone overdependence was 2.670 times higher (OR = 2.670; 95% CI = 1.327, 5.375) for “somewhat” and 4.453 times higher (OR = 4.453; 95% CI = 2.122, 9.346) for “very” when compared to “not at all.” The subjective evaluation of sleep quality (the extent to which respondents recovered from fatigue by sleeping) was also significantly associated with smartphone overdependence. The group was strongly negatively associated with a lower of being “Neither sufficient nor insufficient” (OR = 0.344; 95% CI = 0.203, 0.583), in comparison to “Insufficient.” Lastly, concerning the experience of loneliness, the likelihood of smartphone overdependence was 2.153 times higher (OR = 2.153; 95% CI = 1.014, 4.570) for “often” and 8.149 times higher (OR = 8.149; 95% CI = 2.651, 25.052) for “Always” answers compared with “Never” answers shown in Figure 1.

**Table 3:** Multinomial logistic analysis results in the mental health variables of the smartphone overdependent group.

**Variables**		**B**	**S.E**	**Wald**	**Odds ratio (95% CI)**
Subjective health status	Good				1 [reference]
	Fair	−0.189	0.274	0.476	0.828 (0.484–1.416)
	Poor	0.133	0.431	0.096	1.143 (0.491–2.661)
Subjective perception of happiness	Happy				1 [reference]
	Neutral	0.605	0.256	5.606	1.831^*^ (1.110–3.022)
	Unhappy	0.879	0.433	4.111	2.408^*^
					(1.030–5.632)
Subjective perception of stress	Not at all				1 [reference]
	Somewhat	0.982	0.357	7.576	2.670^**^ (1.327–5.375)
	Very	1.494	0.378	15.591	4.453^***^ (2.122–9.346)
Sadness and despair	No				1 [reference]
	Yes	−0.376	0.255	2.169	0.687 (0.417–1.132)
Subjective evaluation of sleep quality	Insufficient				1 [reference]
	Neither sufficient nor insufficient	−1.067	0.269	15.689	0.344^***^ (0.203–0.583)
	Sufficient	−0.388	0.272	2.03	0.679 (0.398–1.157)
Experience of loneliness	Never				1 [reference]
	Rarely	0.208	0.352	0.35	1.232 (0.618–2.456)
	Sometimes	0.122	0.313	0.153	1.13 (0.612–2.086)
	Often	0.767	0.384	3.985	2.153^*^ (1.014–4.570)
	Always	2.098	0.573	13.406	8.149^***^ (2.651–25.052)

*** *p* < 0.001,

** *p* < 0.01,

* *p* < 0.05,

S.E, Standard Error; CI, confidence interval; Group 1, Smartphone non-dependent group (lower than 10 points); Group 2, Smartphone overdependent group (37 points or higher), the Reference category is group 1.

## Discussion

As smartphone adoption and use increase, smartphone overdependence has manifested worldwide. Smartphone overdependence in adolescents causes indiscriminate exposure to information, disruption in learning involvement, and decreased physical activity (18). Hence, not only does it have a negative impact on balanced growth and development, but it is also highly likely to create a societal problem as smartphone overdependence during adolescence is strongly linked to smartphone addiction in adulthood (10, 11). Accordingly, this study aimed to investigate the relationship between smartphone dependency and the mental health of adolescents and to provide basic data needed in developing measures for the prevention of smartphone overdependence in order to promote healthy growth. The study findings showed that all mental health factors were significantly different according to smartphone dependency level and that subjective perceptions of happiness and stress, sleep satisfaction, and the experience of loneliness was significantly influenced by smartphone overdependence.

The results of the present study clearly support the strong association in mental health factors according to smartphone dependency level that all mental health variables showed statistically significant differences (*p* < 0.001). Specifically, the proportions of respondents whose subjective health status was poor and who felt unhappy were higher in the smartphone over-dependent group than in the smartphone non-dependent group. Additionally, the proportions of respondents experiencing high-stress levels, sadness/despair, and loneliness were higher in the former group. These findings are in line with previous study findings that adolescent smartphone addiction has a negative impact on mental health (20, 21) and that adolescents with greater smartphone overdependence proneness are more likely to show depressive symptoms (22). It is believed that the government needs to take aggressive measures to prevent and manage mental health problems in adolescents showing smartphone overdependence.

The smartphone over-dependent group had a lower proportion of respondents that their sleep quality is sufficient than the smartphone non-dependent group did. This finding is also consistent with previous findings that heavy smartphone use was also directly associated with poor sleep quality and delayed sleep patterns (23) and that college students with smartphone over-dependence disposition had low-quality sleep (24, 25). Because, during adolescence, sleep is an important factor affecting growth, academic achievement, and mental health (26), interventions should be developed to educate adolescents who are over-dependent on smartphones to sleep for a sufficient duration.

In addition, this study was mainly designed to investigate the association between smartphone dependency and mental health factors in adolescents showed that subjective perceptions of happiness and stress, sleep satisfaction, and the experience of loneliness was statistically significantly associated with smartphone dependency (*p* < 0.05). Specifically, the more Korean adolescents felt unhappier and the more they felt stressed, the higher the likelihood of their smartphone overdependence. The likelihood decreased as adolescents were unsatisfied with their sleep quality, but increased as they more frequently felt lonely. These findings are consistent with previous study findings that increased smartphone use in adolescents is linked to physical and psychological health problems, like low sleep quality (27), and resulted in feelings of loneliness (28). In the current study, compared to the smartphone non-dependent group, in the smartphone over-dependent group, happiness, stress, sleep, and loneliness had negative impacts.

In modern society, smartphone use is closely linked to daily life overall (29). Among adolescents, a phenomenon has emerged in which they are dependent on smartphones so as not to be isolated from peers (30) and to be emotionally secure (31). But, someone who is addicted to a smartphone will feel happy when using a smartphone, then feel stressed and anxious when they can't meet the needs of using their smartphone (32). One study found a negative direct association between satisfaction with life and smartphone use in one of their samples (33).

In addition, smartphone overdependence makes it difficult to develop healthy sleep habits (34) and prevents users from having high-quality sleep owing to the blue light emitted by the smartphone screen even during sleep hours (35). Adolescents' reasons for basic smartphone ownership were largely focused on their desire to be connected to their social networks (36). But, adolescents are used to communicating through smartphones in a manner limited to visual and auditory perceptions during the phase in their lifespan in which sociability and emotions should be developed in diverse situations, and smartphone use gives them fewer opportunities to develop solid interpersonal relationships. As adolescents find real-life communication difficult, they feel empty and socially isolated owing to the interpersonal relationships being formed that are detached from physical reality, and thus, smartphone overdependence is predicted in adolescents who frequently feel lonely (37, 38). Because mental health in adolescents is closely linked to smartphone overdependence, as shown above, aggressive measures are needed to help them develop desirable life habits by appropriately controlling smartphone use. In developing such measures, realistic approaches to widely pervasive smartphone overdependence among adolescents should be explored by taking into account mental health factors, that is, predictors of smartphone overdependence.

This study had positive strengths, first, it used national statistical data to define smartphone overdependent and smartphone non-dependent, and second, it had meaningful in that it examined the association between smartphone dependence and mental health factors in adolescents with age- and gender-matched populations. However, there are a few limitations of the study. First, owing to the cross-sectional study design, we were unable to identify the tendency relation between smartphone dependency and mental health. Second, the study could not more detail identify other levels of smartphone dependence because it compared and analyzed low and high smartphone-dependent groups. Third, it was not possible to consider various factors that could affect the smartphone dependency of adolescents, such as changes in smartphone use, lifestyle, and the home environment caused by COVID-19.

## Conclusion

This study examined differences in mental health variables according to smartphone dependency levels in adolescents. These findings provide a greater understanding of the mental health factors predicting smartphone overdependence, subjective perceptions of happiness, stress, sleep satisfaction, and loneliness negatively influenced overdependence. The results of these studies suggested that teenagers can acquire information through smartphones, form social relationships, and try to relieve stress, but it has a negative impact on actual emotional satisfaction. Therefore, rather than unconditionally restricting adolescents from using smartphones, parents and the government should pay attention to exploring using smartphone control levels and techniques appropriate for adolescents, and they are needed to develop a variety of educational methods to develop healthy adolescents. In order to prevent adolescents' dependence on smartphones, parents need to mutually discuss the use of smartphones, have high-quality conversations, and show exemplary smartphone usage behavior to adolescents. In addition, education authorities will be encouraged to develop and use applications to help them use smartphones correctly, conduct a survey on the use of smartphones after COVID-19, and conduct educational campaigns on how to form peer relationships and use leisure time instead of using smartphones.

## Data availability statement

The raw data supporting the conclusions of this article will be made available by the authors, without undue reservation.

## Ethics statement

The studies involving human participants were reviewed and approved by the study used anonymized and deidentified data on the sixteenth KYRBS as a government-approved statistical survey (Approval number: 117058). Written informed consent to participate in this study was provided by the participants' legal guardian/next of kin.

## Author contributions

N-HK and S-HY: data curation. N-HK and Ju-ML: formal analysis. Ja-ML and S-HY: investigation. Ju-ML and Ja-ML: methodology. Ju-ML: project administration. N-HK: writing—original draft. S-HY and Ju-ML: writing—review and editing. All authors have read and agreed to the published version of the manuscript.
